# 靶向于癌症治疗的信号通路

**DOI:** 10.3779/j.issn.1009-3419.2012.09.10

**Published:** 2012-09-20

**Authors:** William CS CHO, 燕 丁

**Affiliations:** 1 Department of Clinical Oncology, Queen Elizabeth Hospital, Hong Kong; 2 天津医科大学总医院，天津市肺癌研究所，天津市肺癌转移与肿瘤微环境重点实验室; 3 香港特别行政区 伊利沙伯医院 临床肿瘤科

**Keywords:** 癌症, EGFR, 信号通路, 靶向治疗, VEGF

癌细胞以存在多种遗传上的改变为特征，这些变异的累积驱动正常细胞向侵袭性癌症进展。在癌变过程中，正常细胞转变为肿瘤细胞至少必须四到六种基因突变^[1]^。近年来，大量遗传学研究以及基于新一代测序技术的全基因组测序研究丰富了我们对癌症进展中分子机制复杂性的认识^[2]^。研究人员已详尽分析了癌症标志的概念，为描述癌症的复杂生物学提供了有用的概念性框架^[3]^。以通路为基础的功能实验研究亦致力于识别癌症中不同信号通路的潜在癌变，这或可为更有效的治疗及更好的效果铺砌一条道路^[4]^。

遗传变异的分子系统性研究或有助于改善我们对癌细胞内信号通路网络如何运作的了解。一项有关胰腺癌的综合性遗传学分析鉴定了63种遗传变异，由这些变异确立了12个核心细胞信号通路，该12个核心信号通路在67%-100%的肿瘤中存在遗传变异^[5]^。亦有研究分析斯坦福微阵列数据库（Stanford Microarray Database）中的人类肿瘤数据，建立了一个功能性癌症图，该图可将数以百计的基因表达谱转化为相应的肿瘤特异性通路活性谱。同样地，研究发现与肿瘤进展相关的多种通路是大多数肿瘤的共同特征。通过在通路活性水平对比肿瘤，该功能性癌症图或可提供不同癌症类型间分子相似性的系统观点^[6]^。

癌细胞基因组的突变影响了在细胞生长、增殖、血管新生、存活、凋亡和转移中具有关键作用的信号通路。这些通路的激活可引起转录因子的上调，诱导细胞中的上皮-间质转化^[7]^。有些信号通路对于胚胎发育至关重要，而胚胎发育在不同人类癌症的肿瘤进展和对治疗反应的变化中发挥关键作用，如Hedgehog和Wnt通路^[8]^。而有些通路的激活以及其相互作用则可增加克服耐药性的难度，如EGFR和VEGF通路。由于缺少对致癌通路和药物抵抗的控制，有效的治疗方法仍较为缺乏。了解活化的信号通路如何提高肿瘤细胞的存活至关重要，因为这或可诱导产生靶向于化疗耐药群组的更有效治疗方式^[9]^。因此，除了识别癌症中的遗传变异，一些研究已聚焦异常调节的信号通路及其在癌症生物学的作用以及其对治疗的反应。了解致癌信号通路及其机制或可为研究癌症的治疗策略提供新的机遇。

在过去的十年里，靶向治疗通过特异性失活肿瘤细胞生长所需的通路革新了癌症治疗^[10]^。因为信号通路在癌症形成和发展中发挥突出作用，大量研究致力于研发靶向作用于分子改变进而干扰癌症特异性相关的信号通路的药物^[11]^。其靶点包括血管新生因子、细胞表面受体、循环生长因子以及组成下游细胞内信号通路的其它分子^[12]^。近年来，一些用以抑制信号通路中多靶点的治疗药物已经涌现。这些多靶点药物同时阻断数个信号通路，可在多种肿瘤类型中发挥作用^[13]^。例如，舒尼替尼是一种多靶点受体赖氨酸激酶抑制剂，在治疗肾细胞癌的过程中可同时抑制VEGF和PDGF通路^[14]^。索拉非尼则是一种同时针对B-Raf、C-Raf、VEGFR和PDGFR的多靶点激酶抑制剂，在晚期肝细胞癌的治疗中具有抗血管新生和抗增殖的作用^[15]^。一项新近研究在三种EGFR信号通路模型中检验了在同一通路中应用两种药物作用于两种蛋白质激酶靶标是否可产生协同作用。有趣的是，正面效应、负面效应或无效应均可观察到，这取决于靶向作用的蛋白激酶及所采用的通路模型。然而，他们的研究结果表明了在同一通路中联合不同的靶向药物用以影响分子信号的可能性^[16]^。

另一方面，相同的靶向因子可涉及一个以上的信号通路。例如对于肿瘤血管新生来说，TGF-β和VEGF信号通路都十分重要。靶向作用于这两个信号通路或可为癌症患者抗血管新生治疗的发展提供新的机会^[17]^。事实上，研究发现多个信号通路在肿瘤发生中发挥重要作用。然而，癌症的遗传异质性以及微环境的动态变化为设计作用于多种通路的药物增加了困难和挑战。一项新近研究研发了一种可双重靶向于Waldenstrom巨球蛋白血症中mTOR和PI3K通路的新型抑制剂NVP-BEZ235。该抑制剂通过直接靶向于肿瘤克隆以及间接通过对骨髓环境的作用发挥对癌细胞的毒性作用^[18]^。通过联合不同的靶向药物抑制多种通路也备受关注，EGFR-VEGF通路相互作用的广泛度预示联合靶向作用于这两个通路的可行性，但仍需研发可抑制自分泌和细胞内信号的化合物^[19]^。同时亦应寻找有效的功能性生物标记物，以识别对双重通路抑制有反应的患者群组^[20]^。

近年来一些关于肿瘤启动和进展的复杂癌症信号通路已被阐明。对信号通路逐步深入的了解正驱动靶向于特定分子事件的新一代抗癌治疗的发展。由于信号通过蛋白激酶的转播而沿着癌症通路进行传播，研发靶向于特定通路的蛋白激酶抑制剂或可为癌症干预构建新的治疗策略。

本专集讨论了在癌症治疗中一些调节异常信号通路的特点和靶标。荟萃了一些为核心癌症信号通路带来新观点的综述性文章，包括有关VEGF、EGFR、IGF1R、Hedgehog、p53、Fas/FasL、MAPK、PI3K以及胚胎等课题，力求捕捉目前围绕癌症信号通路的亮点研究。鉴于该领域的快速发展，众多有关通路研究的新进展已被选录。我们期望它可为癌症信号通路研究的新发现以及基于通路治疗的研发提供最新指导。这些新发现和治疗进步或可促进各种癌症的临床治疗的长足进步。
